# Identifying Personal Values Influencing an Older Adult’s Lifestyle: Insights from Relative Importance Analysis Using Machine Learning

**DOI:** 10.1002/alz.086046

**Published:** 2025-01-09

**Authors:** Seungju Lim, Ji‐Hyuk Park

**Affiliations:** ^1^ Yonsei University, Wonju, Gangwon‐do Korea, Republic of (South)

## Abstract

**Background:**

There is a growing global interest in the healthy lifestyle of older adults. However, despite lifestyle representing the individual, their way of life, research focusing on their personal values in the influence of lifestyle factors is lacking. This study aims to categorize the lifestyles of older adults into two types and utilize machine learning to identify the personal values that take precedence influence these lifestyles.

**Method:**

This study is a cross‐sectional study targeting middle‐aged and older adults aged 55 and above living in local communities in South Korea. The study utilized data from 300 participants, collected through online surveys. The lifestyle types were dichotomized by YLP‐ABCD (Yonsei Lifestyle Profile ‐ Active, Balanced, Connected, Diverse) responses using latent profile analysis. The personal value information was collected by YLP‐V (Yonsei Lifestyle Profile ‐ Value) and analyzed by machine learning to identify the relative importance of the personal values on their lifestyle types.

**Result:**

The lifestyle of older adults was categorized into a healthy lifestyle type (48.87%) and an unhealthy lifestyle type (51.13%). These two types showed the most significant difference in social relationship characteristics. The healthy type demonstrated well‐connected and active social interaction with people, while the unhealthy type exhibited the opposite. Among the machine learning models included in this study, the model that demonstrated the highest classification performance was the support vector machine, with an accuracy of 96% and an Area under the ROC Curve of 95%. Using this model, the relative importance of personal values results showed that individuals with a strong interest in maintaining a healthy diet, curiosity about health information, and engagement in hobbies and cultural activities were more likely to belong to the healthy lifestyle type.

**Conclusion:**

The findings highlight the importance of a personal value‐centered approach in managing the healthy lifestyle of older adults, indicating that an approach emphasizing active social relationships, engagement in diverse activities, and an interest in a healthy diet is necessary.

